# Real-time biofluorescent particle counting compared to conventional air sampling for monitoring airborne contamination in orthopedic implant surgery

**DOI:** 10.1017/ash.2025.61

**Published:** 2025-04-07

**Authors:** Frans Stålfelt, Josefin Seth Caous, Karin Svensson Malchau, Camilla Björn, Maziar Mohaddes

**Affiliations:** 1 Department of Orthopedics, Institute of Clinical Sciences, Sahlgrenska Academy, University of Gothenburg, Gothenburg, Sweden; 2 Division Materials and Production, Methodology, Textiles, and Medical Technology, RISE Research Institutes of Sweden, Gothenburg, Sweden; 3 Department of Clinical Sciences Lund – Orthopedics, Lund University, Lund, Sweden; 4 Department Orthopedics, Hässleholms Hospital, Hässleholm, Sweden

## Abstract

**Background:**

Surgical site infection (SSI) following orthopedic surgery is a complication associated with morbidity and economic burden. Transmission of airborne bacteria that settle into surgical wounds constitutes a risk factor for SSIs. However, monitoring microbial contamination inside operating rooms with conventional methods is resource and time-consuming.

**Aim:**

This study aimed to assess correlation between a biofluorescent particle counter (BFPC) and conventional air sampling, to enable real-time monitoring of airborne contamination. Additionally, the study aimed to analyze correlation between particles near the surgical site and particles 1 meter away, to evaluate the feasibility of distance-based measurements.

**Methods:**

Correlation analysis was conducted to compare colony-forming units (CFU) collected using a Sartorius MD8 air sampler with biofluorescent viable particles detected by BioTrak 9510-BD, both positioned near the surgical site. Additionally, correlation between particle counts measured by AeroTrak 6510, positioned 1 meter away, and total particle counts measured by the BioTrak near the surgical site was evaluated. Sampling took place in two operating rooms: one with turbulent mixed airflow (TMA) and one with unidirectional airflow (UDAF).

**Results:**

Negligible to low correlation between biofluorescent particles and CFU was observed, both in UDAF (n = 100) and TMA (n = 22). However, strong correlation was found between BFPC and particle counter measurements of total numbers of particles (R_p_ = 0.634–0.769, *P* < .001).

**Conclusion:**

While BFPCs offer real-time monitoring of airborne contamination, their predictive ability for CFU levels remains uncertain. Yet, the strong correlation between particles in the surgical site and particles measured 1 meter away suggests feasibility to conduct future studies with larger cohorts.

## Introduction

Surgical site infection (SSI) after orthopedic implant surgery constitutes a serious complication,^1^ where the incidence rates range from 0.76% to 1.24% for total hip arthroplasty (THA) and 0.88% to 1.28% for total knee arthroplasty (TKA).^2^ Additionally, SSI leads to prolonged patient suffering and substantial economic ramification, imposing significant societal costs and necessitating additional healthcare resources.^3^ While the precise infectious pathways remain largely elusive, one possible way for bacteria-carrying particles to contaminate the surgical wound is via airborne transmission,^4–7^ either directly or indirectly via surgical instruments or implants.^8,9^ Bacteria are typically sized between 1 and 2 µm per cell however often clustering together to form larger aggregates, which together with the carrying fragment of eg skin define the total size of the particle.^6^ Identified risk factors for particle emission include the shedding of skin fragments from staff, door openings, and the type of clothing worn in operating rooms (ORs).^10,11^ This measure, complemented by other preventive actions, such as antibiotic prophylaxis, proper hygienic protocols, and skin preparation, are important to minimize the risk for SSI.^12^

The conventional method for microbial air assessments in ORs involves active air sampling, where airborne bacteria are captured on agar plates, followed by incubation and quantification of colony-forming units (CFU).^13,14^ While effective for detecting bacterial contamination near the surgical site, active air sampling is challenging to perform due to its intrusive equipment, which can distract surgeons.^15^ Additionally, obtaining results is both time- and resource-intensive, and requires specialized microbiological skills, often necessitating additional staff in the OR. In contrast, industries employing cleanroom technologies have long relied on automated real-time particle monitoring systems to maintain minimal levels of particulate contamination.^16,17^ Despite similarly rigorous policies regarding airborne contamination in ORs, the widespread implementation of such systems in healthcare remains limited. This hesitation is primarily attributed to the lack of robust evidence linking particulate levels to CFUs.^14^ Establishing evidence could enable real-time surveillance in ORs, facilitating timely interventions to reduce microbial contamination.^4,6,18,19^

In a previously published systematic review examining the relationship between particulate levels and CFUs, the yielded results were mixed, making it difficult to establish a definitive correlation.^20^ While most prior research in ORs has utilized optical particle counters (PCs) to explore the association between total particulate levels and CFUs, modern biofluorescent particle counters (BFPCs) offer a promising alternative to detect viable particles. Although BFPCs have significant potential for microbial surveillance,^21–24^ correlation between biofluorescent particles and CFU have not been extensively studied in clinical settings. A study by Dai et. al explored this relationship and found a significant correlation (Pearson’s correlation coefficient = 0.76). However, the limited sample size from this study emphasizes the need for further research to validate these findings during ongoing surgeries.^25^

The aim of this study was to assess the correlation between biofluorescent particles (BFP) and CFU during orthopedic implant surgeries, to facilitating real-time monitoring of air bacterial contamination during surgical procedures. A secondary aim was to analyze the correlation between total particle counts measured by BFPCs near the surgical wound and total particle counts obtained by a conventional optical PC positioned 1 meter away, to assess the feasibility of accurate measurements at greater distances for future studies.

## Method

### Study information

This observational investigation took place during the spring of 2022 at Sahlgrenska University Hospital, Mölndal, involving two ORs for orthopedic implant surgery; one equipped with vertical unidirectional airflow (UDAF) ventilation and the other with turbulent mixed airflow (TMA) ventilation. Eligible surgical procedures performed on weekdays were included in the study after approval by the lead scrub nurse. The study included THAs and TKAs performed in the UDAF-ventilated OR, as well as hip hemiarthroplasties (HHAs) conducted in the TMA-ventilated OR. Air sampling procedures adhered to the Swedish Institute of Standards (SIS) technical specification (TS) SIS-TS 39:2015.^14^ The OR team consisted of a lead surgeon, an assistant surgeon, a scrub nurse, an assisting nurse, and an anesthetic nurse. All staff wore surgical attire made of a 50/50 cotton/polyester blend, along with tucked-in surgical hoods. Additionally, the surgeons and the scrub nurse wore disposable non-woven surgical gowns (Mölnlycke, Barrier Surgical Gown), double sterile gloves, and face masks. Ethical approval for the study was granted by the Swedish Ethical Review Authority (2021–01689), and the study is registered on ClinicalTrials.gov (NCT05816135).

### Materials and instrumental setup

**Figure 1.**
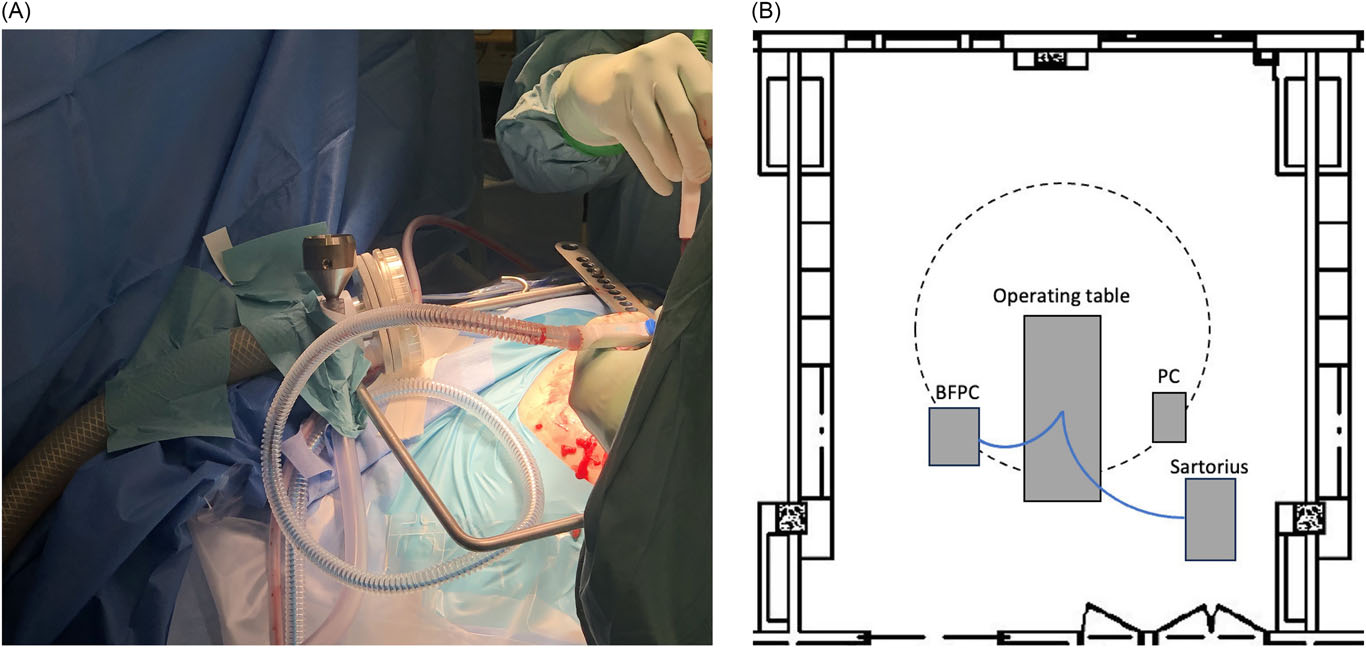
**A)** Instrumental setup showing the biofluorescent particle counter probe alongside the tube and mouthpiece of the Sartorius MD8 air sampler, equipped with a gelatin membrane filter. **B)** Schematic of the instrument placement inside the operation room (OR) with vertical unidirectional airflow (UDAF) ventilation. The circled area indicates the aerial safe zone generated by the vertical UDAF. Similar instrumental placement was used in the turbulent mixed airflow ventilated OR, with the exception that no additional particle counter was used.

Bacterial samples were acquired using the active air sampler Sartorius MD8 (Sartorius Lab Instruments GmbH & Co. KG, Göttingen, Germany) operating at a flow rate of 100 L/min for a duration of 10 minutes, equivalent to 1 m^3^/10 min. A sterilized tube (PVC, L = 1.5 m, ⌀ = 10 mm) was affixed to the Sartorius MD8, positioned approximately 0.3 m from the surgical wound (Figure 1A). A sterilized mouthpiece was fixated to the tube’s end, where the filtered air samples impinged upon gelatin filters (Sartorius Lab Instruments GmbH & Co. KG) featuring pore sizes of 3 µm. Following the collection of 1 m^3^ of filtered air, the gelatin filter was aseptically detached by the scrub nurse and handed to a trained professional proficient in environmental sampling techniques, who then transferred the filter to nonselective horse blood agar plates (Substrate Department, Sahlgrenska University Hospital) as recommended by SIS-TS 39:2015.^14,26^ Post-surgery, the samples were taken to the biosafety level 2 research laboratory at the Department of Orthopedics, Sahlgrenska University Hospital and incubated at 35 ± 2 °C and inspected after 2 and 5 days for CFU count.^14,26^ Depending on the duration of the procedure (typically 45–70 minutes), 4–6 air samples were collected from incision to wound closure. The results for each air sample were expressed as colony-forming units per cubic meter (CFU/m³). Identification of bacterial types was not performed.

Viable particle count was acquired utilizing the BFPC BioTrak 9510-BD (TSI, Minnesota, USA). The BFPC was connected to a sterilized polyvinyl chloride tube (L = 1.5 m, ⌀ = 8 mm) and positioned in close proximity to the headpiece of the Sartorius MD8 (Figure 1A), in order to ensure comparable samples. The configuration follows the manufacturer’s recommendations and complies with international standard guidelines.^14,17,27^ Additionally, the setup was verified in consultation with a cleanroom measurement expert to ensure accuracy and adherence to best practices. Air sampling was conducted continuously throughout the surgical procedure, from the initial incision to the closure of the surgical wound, to evaluate the clinical applicability of the sampling method. The BFPC collected samples at a rate of 0.0283 m^3^/min, with data logged at one-minute intervals. For the analysis, the collected data used was the mean value to match the 10-minute interval of the Sartorius MD8. Results were automatically converted into the unit of number of particles per cubic meter (m^3^) for comparability with the results from Sartorius MD8. The BFPC detected particle sizes of 0.5, 0.7, 1, 3, 5, and 10 µm, both viable and total count. However, particle sizes of 0.5 and 0.7 µm were excluded from the analysis due to smaller particles being less likely to carry bacteria.^6^ The BioTrak aggregates particle counts for all particles equal to or greater than a defined size threshold, with the reported count reflecting the total number of particles within the specified size range and above.

A conventional PC, AeroTrak 6510 (TSI, Minnesota, USA), was positioned 1 meter away from the surgical table, within the periphery of the surgical zone (Figure 1B). The AeroTrak operated at a flow rate of 28.3 L/min and employed photodetection for particle count analysis. In contrast to the BioTrak 9510-BD, this particle counter only detected particles of sizes 0.5, 0.7, 1, and 5 µm. Particle sizes were compared consistently with the respective particle channel for the PC and the BFPC (1 and 5 µm). Continuous data sampling was systematically executed from the incision phase to the surgical wound closure, the same as the protocol employed for the BioTrak. Notably, data of conventional particle counting was only accessible for UDAF ventilation, due to the AeroTrak being already installed in this OR.

### Statistical methods

The statistical analysis was conducted utilizing IBM SPSS Statistics version 29.0.0.0. The particle measurements were divided into time intervals corresponding to the CFU measurements, with mean values used for both the BFPC and the conventional PC for the correlation analysis. To facilitate the interpretation of the descriptive analysis, the particulate data were transferred into a logarithmic scale. Pearson’s correlation coefficient (R_p_) was applied for parametric data. Assumptions were tested using the Shapiro-Wilk test and via visualization through distribution- and QQ-plots. Correlations were ranked accordingly: Negligible correlation (R < 0.1), weak correlation (0.1 ≤ R ≤ 0.39), moderate correlation (0.4 ≤ R ≤ 0.7), and strong correlation (R > 0.7).^28^

## Results

### Sample collection and characteristics

In total, the study observed 22 surgical procedures, including THA (n = 11), TKA (n = 7) and HHA (n = 4). THA and TKA procedures were conducted in an OR equipped with UDAF ventilation, while HHA was performed in an OR utilizing TMA ventilation. A total of 122 paired samples of CFU and BFP were collected from the two ORs, with a distribution of 100 samples in UDAF and 22 in TMA. Across all surgical procedures and ORs, the concentration of smaller airborne particles consistently exceeded that of larger particles. Notably, the mean CFU/m^3^ was significantly higher in samples collected from TMA-ventilated ORs compared to UDAF-ventilated ORs, as shown in Table 1.

**Table 1.** Descriptive statistics of colony-forming unit (CFU) and logarithmic particle levels of all measured particle sizes

		UDAF (n = 100)		TMA (n = 22)	
Variable	Sample location	Median	IQR	Median	IQR
CFU/m^3^	Surgical site	1.3	0–2.0	6.5	4.0–11.0
log_10_ BFP/m^3^	Surgical site				
1 µm		3.21	2.92–3.64	3.38	3.17–3.62
3 µm		2.60	2.30–2.97	2.92	2.64–3.18
5 µm		2.07	1.77–2.39	2.30	2.13–2.49
10 µm		1.24	1.00–1.47	1.55	1.24–1.69
log_10_ total particles/m^3^	Surgical site				
1 µm		4.25	3.91–4.76	4.23	4.06–4.44
3 µm		3.33	3.00–3.66	3.64	3.32–3.81
5 µm		2.96	2.61–3.25	3.25	3.00–3.41
10 µm		2.32	2.08–2.59	2.82	2.56–2.92
log_10_ particles/m^3^	1 meter from surgical site				
1 µm		4.15	4.01–4.34	*	*
5 µm		3.04	2.88–3.19	*	*

IQR, interquartile range.

* Not applicable.

### Correlation between BFP, PC and CFU in UDAF and TMA

Correlation analysis between the measured concentrations of CFU and BFP of sizes 1 µm, 3 µm, 5 µm, and 10 µm, in UDAF-ventilated OR showed an overall negligible to weak correlation, with no statistical significance. Similarly, a negligible correlation was observed between particles of sizes 1 µm and 5 µm measured by the PC, and CFU. In TMA-ventilated operating rooms the correlation analysis of BFP/m^3^ and CFU/m^3^ showed week correlation for all particle sizes, as seen in Table 2.

**Table 2.** Correlation coefficients in unidirectional airflow (UDAF)-ventilated operation room (OR) (n = 100) and turbulent mixed airflow (TMA)-ventilated OR (n = 22) between colony-forming unit (CFU) and BFP and particle counter (PC), and between total particle count measured by the biofluorescent particle counter (BFPC) and the PC

Ventilation	n	Comparison	Particle size (µm)	Correlation coefficient	*P*-value
UDAF	100	BFP vs CFU	1	0.107 (–0.091–0.297)	.288
			3	–0.068 (–0.261–0.130)	.501
			5	0.132 (–0.066–0.320)	.132
			10	0.061 (–0.137–0.255)	.545
		PC vs CFU	1	–0.004 (–0.201–0.192)	.966
			5	0.042 (–0.156–0.237)	.667
		BFPC^a^ vs PC	1	0.769 (0.674–0.839)	<.001
			5	0.634 (0.500–0.738)	<.001
TMA	22	BFP vs CFU	1	0.090 (–0.345–0.493)	.691
			3	0.175 (–0.266–0.556)	.435
			5	0.259 (–0.183–0.613)	.245
			10	0.211 (–0.231–0.581)	.347

Results are displayed as Pearsons’s Correlation Coefficient (95% Confidence Interval).

Correlation significant at the 0.05 level (two-tailed).

a Total particle count.

### Correlation between BFPC and PC in the UDAF-ventilated OR

**Figure 2.**
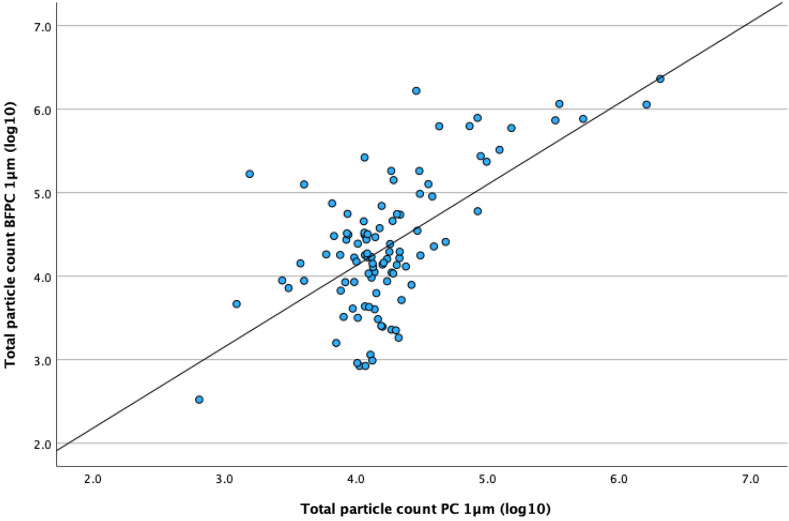
Scatter plot of total count of particles (1 µm) measured with a biofluorescent particle counter positioned close to the surgical site and with a conventional particle counter, positioned approximately 1m from the surgical site in a unidirectional airflow-ventilated OR.

The correlation analysis between total particle counts using BFPC and PC, positioned close to the surgical wound and further away from the operating table, respectively, displayed a strong significant correlation for 1 µm, R_p_ = 0.769, *P* < .001 and a moderate significant correlation for 5 µm, R_p_ = 0.634, *P* < .001, as outlined in Table 2 and visualized in Figure 2.

## Discussion

SSI following orthopedic implant surgery poses significant risks to patient outcomes and healthcare resources. This study evaluated the accuracy of using a BFP to measure airborne bacteria levels during surgery and examined the correlation between CFU and biofluorescent particles BFP under different ventilation conditions. The results indicated a weak correlation between BFP and CFU in both UDAF and TMA-ventilated ORs for all particle sizes. These findings align with the results from a systematic review of the correlation between particles and CFU during ongoing surgery, revealing no discernible evidence for correlation between particles and CFU.^20^ The small sample size in the TMA-ventilated OR likely contributed to the weak and unsignificant correlation between CFU and BFP. Also, the low CFU levels observed in the UDAF-ventilated OR suggests that more samples are needed to facilitate a more robust correlation analysis.

However, mean CFU levels were higher in TMA-ventilated ORs, reinforcing the importance of airflow control, with UDAF systems providing better air quality. This also aligns with previous research indicating that UDAF systems create a more controlled environment with lower bacterial counts due to the establishment of an aerial safe zone over the operating table. Furthermore, the moderate and strong correlation between BFPC and conventional PC suggests that PCs can reliably measure total particle counts even when placed away from the surgical site. This could streamline monitoring without distracting surgeons or requiring additional staff involvement in future research.

The limitations of this study should be considered when interpreting the results. Firstly, the sample size, particularly in TMA-ventilated ORs (n = 22), was small, potentially limiting statistical power and generalizability. The results from the TMA-ventilated OR should therefore be used with great caution. Furthermore, the COVID-19 pandemic led to a reduction in the overall number of surgeries and measurements initially planned for inclusion, with 25 procedures per ventilation setting similar to other comparable studies.^20^ This limitation may have introduced bias by restricting the capture of the full range of operating room conditions. The lack of randomization, where elective surgeries were performed in UDAF-ventilated ORs and trauma surgeries in TMA-ventilated ORs, introduces potential confounders such as variations in door openings, staff presence, and surgery duration, making direct comparison between the two ventilation systems difficult. Additionally, the AeroTrak PC was only used in UDAF-ventilated ORs, restricting comparisons in particle counts between ventilation types. Overall, larger-scale, randomized studies are needed to confirm these findings and provide more robust evidence regarding the impact of ventilation systems on air cleanliness and SSI.

## Conclusion

This study underscores the complexity of airborne bacterial transmission during orthopedic surgeries, emphasizing the influence of ventilation, airflow dynamics, and environmental factors. Controlled environments, such as UDAF-ventilated operating rooms, demonstrated lower bacterial contamination compared to TMA-ventilated ORs. While BFPCs offer real-time monitoring, their correlation with CFU is limited and should be used with caution. However, the strong correlation between total particle counts measured near and further away from the surgical site suggests the potential for less intrusive monitoring. Future improvements in BFPC technology, along with identifying surgical stages that generate high particle counts, could enhance infection control strategies.

## Supporting information

Stålfelt et al. supplementary material Stålfelt et al. supplementary material

## Supplementary material

For supplementary material accompanying this paper visit http://doi.org/10.1017/ash.2025.61. click here to view supplementary material
