# Supplementary material for: Real-time biofluorescent particle counting compared to conventional air sampling for monitoring airborne contamination in orthopedic implant surgery
Source: Antimicrob Steward Healthc Epidemiol. 2025 Apr 7;5(1):e93. doi: 10.1017/ash.2025.61 (PMC11986878; doi:10.1017/ash.2025.61)
Supplement: Stålfelt et al. supplementary material [file S2732494X25000610sup001.docx]

# **Supplementary information**

**OR room properties**

UDAF: The room had a floor area of 51 m² and a total volume of 159 m³. It was supplied with high-efficiency particulate air (HEPA)-filtered air, with a vertical laminar airflow velocity of 0.27 m/s, a supply airflow rate of 760 L/s, and an exhaust airflow rate of 416 L/s.

TMA: The room had a floor area of 40.8 m² and a total volume of 121.7 m³. It was supplied with HEPA-filtered air with an air exchange rate of 21.8 per hour, a supply airflow rate of 560 L/s, and an exhaust airflow rate of 501 L/s.

**Supplementary Table 1.** Additional routine variables, including occurrences of door openings and the count of personnel present in the OR. During the surgical procedures, sensors systematically gathered data concerning environmental parameters within the OR. specifically, temperature. relative humidity, and air pressure differentials relative to the adjoining corridor.

| Parameter | UDAF  (n=18) | TMA  (n=4) | p-value |
| --- | --- | --- | --- |
| Door openings (n) | 1.11 (1.02) | 1.75 (1.5) | 0.309 |
| Staff members (n) | 5.37 (2.63) | 6.20 (0.54) | 0.542 |
| Temperature (°C) | 23.31 (0.19) | 24.58 (4.5) | 0.183 |
| Humidity (%RH) | 23.92 (5.87) | 21.99 (13.59) | 0.611 |
| Diff. air pressure (Pa) | 5.32 (1.86) | 9.98 (0.75) | <0.001* |
